# DNA damage in lens epithelial cells exposed to occupationally-relevant X-ray doses and role in cataract formation

**DOI:** 10.1038/s41598-020-78383-2

**Published:** 2020-12-10

**Authors:** Ion Udroiu, Antonella Sgura, Agnese Chendi, Lorenzo Lasagni, Marco Bertolini, Federica Fioroni, Vando Piccagli, Antonio Moramarco, Maria Grazia Romano, Luigi Fontana, Daniela D’Alessio, Vicente Bruzzaniti, Antonella Rosi, Sveva Grande, Alessandra Palma, Claudia Giliberti, Mauro Iori, Lorenzo Piergallini, Marco Sumini, Lorenzo Isolan, Giorgio Cucchi, Gaetano Compagnone, Lidia Strigari

**Affiliations:** 1 Department of Science, University of Rome “Roma Tre”, Rome, Italy; 2 Medical Physics Unit, Azienda USL-IRCCS di Reggio Emilia, Reggio, Italy; 3 Postgraduate School in Medical Physics, University of Bologna, Bologna, Italy; 4 Postgraduate School in Medical Physics, University of Firenze, Florence, Italy; 5 Ophthalmology Unit, Azienda USL-IRCCS di Reggio Emilia, Reggio, Italy; 6 Department of Medical Physics, St. Orsola-Malpighi University Hospital, Bologna, Italy; 7 Laboratory of Medical Physics and Expert Systems, Regina Elena Cancer Institute IRCCS, Rome, Italy; 8 Istituto Superiore di Sanità, Centro Nazionale Tecnologie Innovative in Sanità Pubblica, Rome, Italy; 9 Inail-Dipartimento Innovazioni Tecnologiche e Sicurezza degli Impianti, Prodotti ed Insediamenti Antropici, Rome, Italy; 10 Montecuccolino Laboratory, Industrial Engineering Department, University of Bologna, Bologna, Italy; 11 INFN, Bologna, Italy; 12 Interdepartmental Center “L. Galvani” CIG, Alma Mater Studiorum Università di Bologna, Bologna, Italy

**Keywords:** Cell biology, Physics

## Abstract

The current framework of radiological protection of occupational exposed medical workers reduced the eye-lens equivalent dose limit from 150 to 20 mSv per year requiring an accurate dosimetric evaluation and an increase understanding of radiation induced effects on Lens cells considering the typical scenario of occupational exposed medical operators. Indeed, it is widely accepted that genomic damage of Lens epithelial cells (LEC) is a key mechanism of cataractogenesis. However, the relationship between apoptosis and cataractogenesis is still controversial. In this study biological and physical data are combined to improve the understanding of radiation induced effects on LEC. To characterize the occupational exposure of medical workers during angiographic procedures an INNOVA 4100 (General Electric Healthcare) equipment was used (scenario A). Additional experiments were conducted using a research tube (scenario B). For both scenarios, the frequencies of binucleated cells, micronuclei, p21-positive cells were assessed with different doses and dose rates. A Monte-Carlo study was conducted using a model for the photon generation with the X-ray tubes and with the Petri dishes considering the two different scenarios (A and B) to reproduce the experimental conditions and validate the irradiation setups to the cells. The simulation results have been tallied using the Monte Carlo code MCNP6. The spectral characteristics of the different X-ray beams have been estimated. All irradiated samples showed frequencies of micronuclei and p21-positive cells higher than the unirradiated controls. Differences in frequencies increased with the delivered dose measured with Gafchromic films XR-RV3. The spectrum incident on eye lens and Petri, as estimated with MCNP6, was in good agreement in the scenario A (confirming the experimental setup), while the mean energy spectrum was higher in the scenario B. Nevertheless, the response of LEC seemed mainly related to the measured absorbed dose. No effects on viability were detected. Our results support the hypothesis that apoptosis is not responsible for cataract induced by low doses of X-ray (i.e. 25 mGy) while the induction of transient p21 may interfere with the disassembly of the nuclear envelop in differentiating LEC, leading to cataract formation. Further studies are needed to better clarify the relationship we suggested between DNA damage, transient p21 induction and the inability of LEC enucleation.

## Introduction

The availability of recent advance in catheter and x-ray imaging has recently boosted the frequency and the introduction of novel and more complex procedures of interventional radiology. The increase of number and typology of these procedures has been accompanied with an increasing concern regarding the occupational exposure to personnel involved as first or second operator for the impact of eye-lens exposure. In fact, the current framework of radiological protection of occupational exposed medical workers reduced the eye-lens equivalent dose limits from 150 to 20 mSv per year (15 mSv for public, apprentices and students between 16 and 18 years old)^1,2^.

Occupational exposure is influenced by many factors including the acquisition parameters (impacting on the dose-rate), the irradiation geometry (affecting the spectrum emerging from the RX tube), the use of radioprotective mobile shielding barriers or protective aprons, as well as the patient size and investigated areas.

All the above parameters affect the incident energy spectrum on the patient as well as the operator eyes being the emerging spectrum substantially degraded from the studied patient/phantom^3^. At photon intermediate energy derived from tube with potential difference in the range 20–100 kV—i.e. in the range relevant to medical diagnostic use—the change from the photoelectric effect to the Compton effect causes a transient decrease of electron energies with a degradation of the incident spectrum. The ionization density is therefore increased and the RBE of low energy X-rays is expected to increase up to 1.3 ÷ 2 when compared to 200 kV X-rays. An RBE higher than unity potentially associated to an increased ionizing radiation risk^4,5^ has been reported for the spectrum of mammography equipment. The fact that the RBE value and the radiation weighting factor for electrons and photons should not always be treated as unity has been suggested also through quantitative evaluation of microdosimetric-kinetic model^6^. For an assessment of the possible difference in effectiveness between scattered and conventional X-rays, the impact on eye-lens has been tested using ad hoc radiobiological studies, and the energy and spectra of incident radiations have been examined using a Monte Carlo simulation with the purpose to better understand the biological results.

In addition, the dose-rate is expected to change in the typical clinical scenario and this decrease could increase the rate of radiation-induced effect.

In this study, biological and physical data are combined to improve the understanding of radiation induced effects on Lens epithelial cells (LEC). In fact, LEC proliferate in the germinative zone at the lens equator and differentiate in the transitional zone, entering the body of the lens and becoming lens fiber cells (without nucleus and organelles). Unrepaired DNA damage is supposed to be the cause of aberrant differentiation of lens fiber cells, subsequently resulting in cataract formation^7^. Indeed, defects in the DNA repair machinery has been proved to be linked to earlier onset of cataract. This has been shown in mice homo- and heterozygous for *ATM*, *BRCA1* and *RAD9*^8^, but also in patients with the well-known DNA repair disorders, such as Trichothiodystrophy, Cockayne, Rothmund-Thomson and Werner syndromes^9^. Thus, DNA damage is, although not the sole, one of main causes of cataractogenesis.

## Materials and methods

### Radiological device

For angiographic procedure INNOVA 4100 (General Electric Healthcare, France) was used (scenario A). All radiological parameters (tube voltage, current, field size, number of frame and time duration) were collected in DOSEWATCH software (General Electric Healthcare, France). The data obtained should be corrected for the attenuation of the couch to get a more accurate estimation of the dose to the investigated phantom. Transmission factors for the couch of several angiographic devices was measured as reported in DeLorenzo et al.^10^.

**Figure 1:**
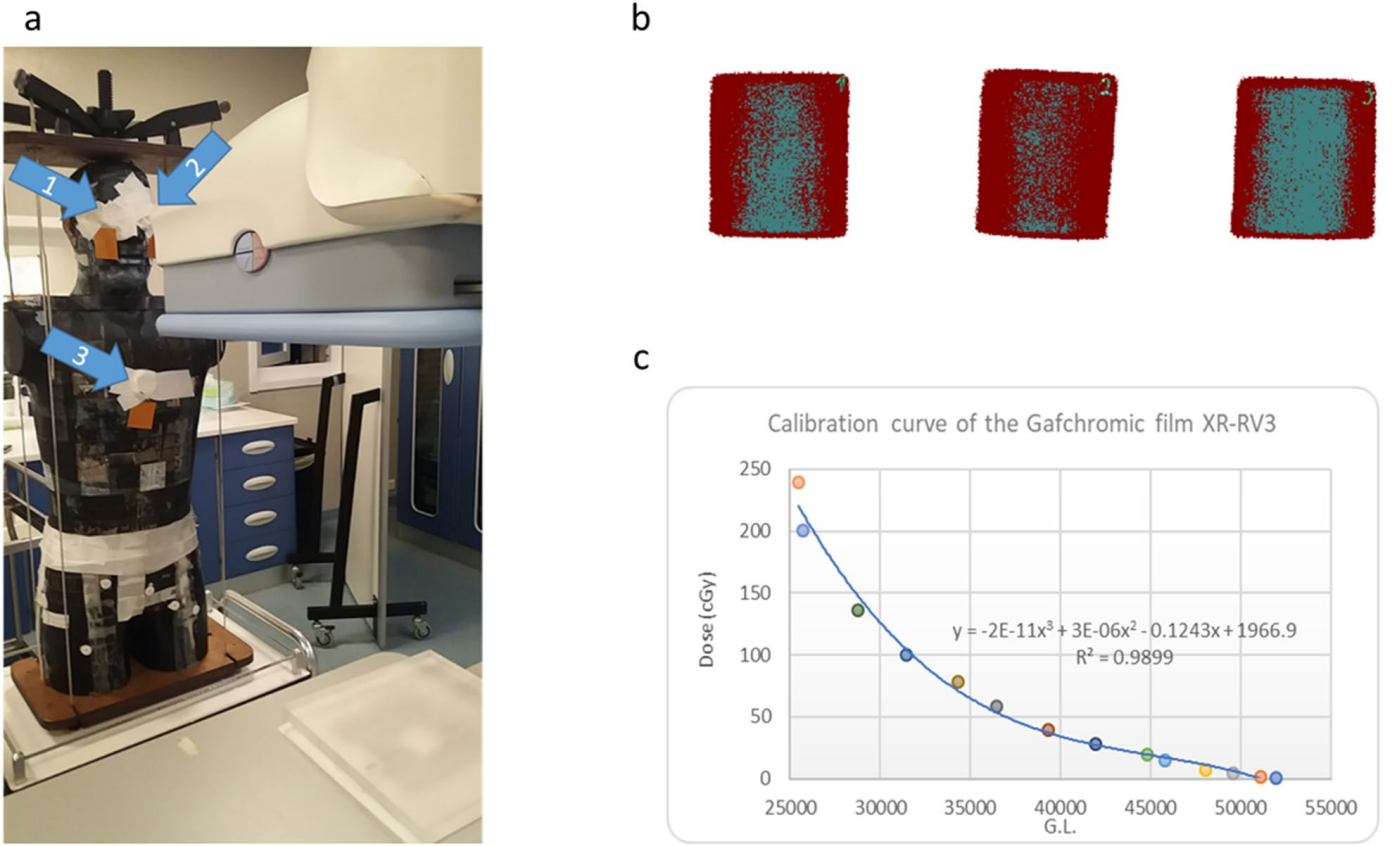
(**a**) experimental setup, (**b**) example of dose map measured using the Gafchromic films XR-RV3 and (**c**) used calibration curve.

The dose was measured using the Gafchromic films XR-RV3 calibrated as described in D’Alessio et al.^11^. The setup and an example of Gafchromic film positions are reported in Fig. 1a, an example of dose map is shown in panel b and the calibration curve is reported in panel c.

In addition, a dedicated Gilardoni (Gilardoni, Italy) radiological equipment (250 kV, 6 mA, 3 mm Al filter) for experimental studies was used at dose rates (DRs) of 0.15 and 0.5 Gy/min (scenario B) already used for conducting independent biological experiments.

### Monte Carlo simulation

**Figure 2:**
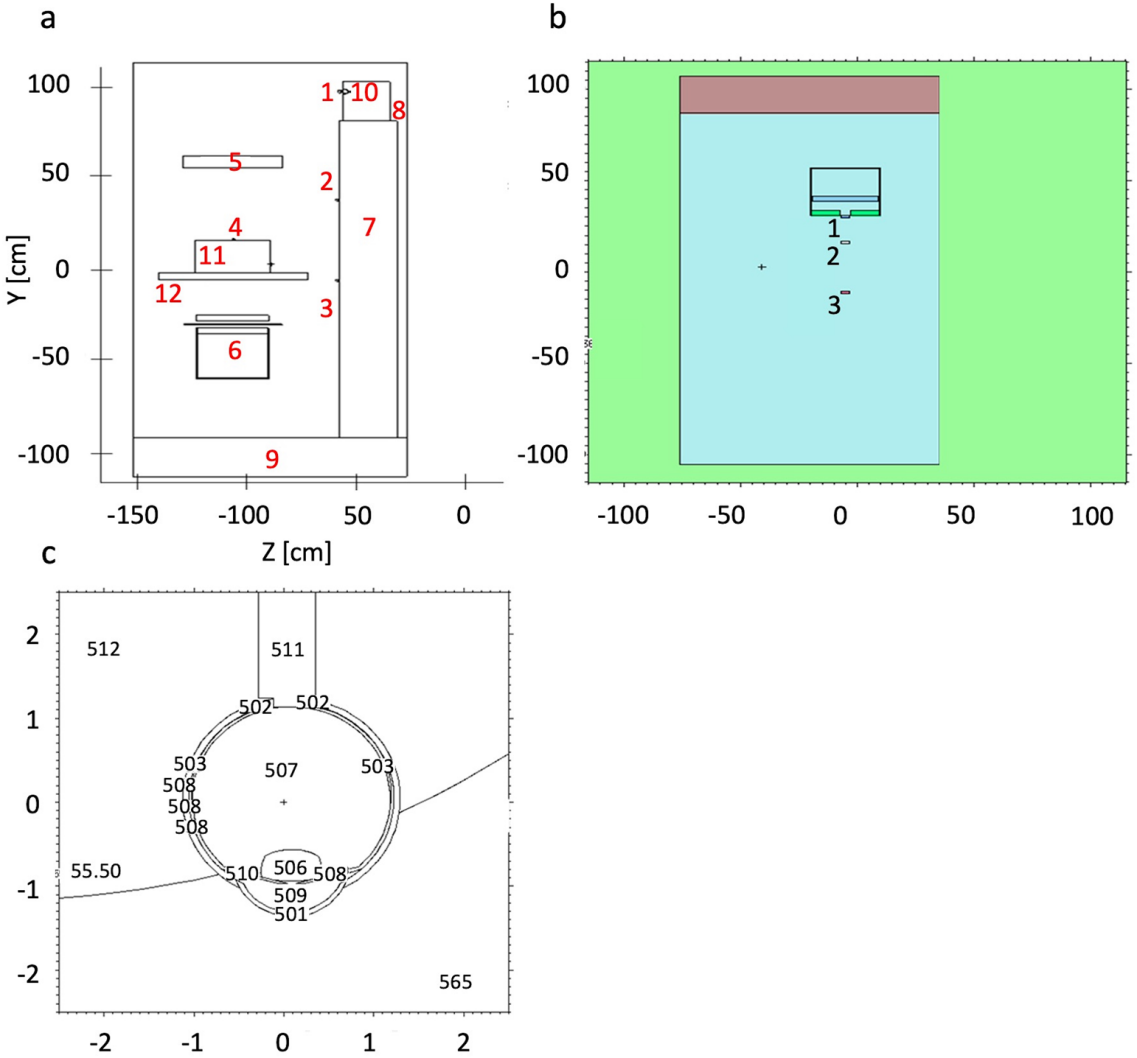
(**a**) Scenario A. MCNP geometry. Left, X-ray exposure setup in Z-Y view; 1, detector simulating the Petri dish at the eye lens level; 2, detector simulating the Petri dish at the chest level; 3, detector for the spectra estimation at the gonads level; 4, detector simulating the Petri dish in the attenuated primary beam; 5, X-ray detector; 6, X-ray system; 7, water phantom simulating the operator body; 8, water phantom simulating the operator head; 9, concrete floor; 10, operator eye (see right part of this figure for details); 11, PMMA phantom; 12, patient couch. (**b**) Scenario B. MCNP geometry visualization. Background color representative of the zero-importance region outside the “simulation box”. The visualized MCNP cells related to the concrete, the X-ray tube model, the Monte Carlo detector and the 2 Petri dishes are shown. Different colors for distinguishing the different MCNP cells. Tally regions 2(B) and 3(B) related to the Petri dish positions. Tally region 1(B) referred to a numerical detector. (**c**) Operator eye in X–Y view as plotted by the MCNP intrinsic viewer. 501, Cornea; 502, Sclera; 503, Retina; 506, Eye lens; 507, Vitreous body; 508, Choroid; 509, Anterior chamber 510, Iris; 511, Optic nerve. Eye model volume, 7.89 cm^3^; Axial length, 2.58 cm; Lens Volume, 0.163 cm^3^; Equatorial length, 0.410 cm; Lens depth, 0.390 cm.

A Monte Carlo study was conducted, in order to support biological results, using a model for the photon generation at X-ray tube energies and with the Petri dishes and numerical detectors placed as described in Fig. 2 (Fig. 2a referred to the A scenario; Fig. 2b referred to the B scenario; Fig. 2c, details of the eye model as implemented in scenario A) and Tables 1, 2, 3, 4, 5. Two different scenarios (A and B) have been considered in order to reproduce the experimental conditions and validate the irradiation setups to the cells, investigating the spectral characteristic of the different beams that have been used for the various photon expositions, calibrating the model against HVL measurements^12,13^. The source spectra have been derived from^14^, as reported by^15^. The simulation results have been tallied using the Monte Carlo code MCNP6 (Monte Carlo N-Particle Transport Code, release 6)^16,17^.

**Table 1:** MCNP6 materials assigned to the eye.

	Crystalline	Vitreous body	Iris	Choroid	Anterior chamber	Cornea	Sclera	Retina	Optic nerve
Material	Eye lens	Water	Water	Water	Water	Tissue (equivalent)	Tissue (equivalent)	Tissue (soft)	Tissue (soft)
Density [g/cm^3^]	1.100000	0.998207	0.998207	0.998207	0.998207	1.000000	1.000000	1.100000	1.100000

**Table 2:** MCNP6 materials assigned to the model.

	X ray tube shield	X-ray tube filter	X-ray tube collimator	Operator’s body	Table	Phantom (patient)	Air	Floor
Material	Lead	Aluminium	Lead	Water	Polyamide	PMMA	Air	Concrete
Density [g/cm^3^]	11.350000	2.699000	11.35000	1.000000	1.420000	1.140000	0.001225	2.250000

**Table 3:** MCNP6 weight fractions of the eye’s materials.

	Water (vitreous)	Eye lens	Soft tissue	Equivalent tissue
ZAID	1nat	6nat	7nat	8nat
Hydrogen	0.111894	0.099269	0.101172	0.101869
Carbon		0.193710	0.111000	0.456179
Nitrogen		0.053270	0.026000	0.035172
Oxygen	0.888106	0.653751	0.761828	0.406780

**Table 4:** MCNP6 weight fractions of the model’s materials.

	H	C	N	O	Ar	Pb			Na	Al	Si	Ca	Fe
ZAID	1nat	6nat	7nat	8nat	18nat	82206	82207	82208	11nat	13nat	14nat	20nat	26nat
Aluminium										1.0000			
Water	0.1119			0.8880									
Polyamide	0.0236	0.6911	0.0732	0.2092									
Lead						0.2429	0.2238	0.5332					
Air		0.0001	0.7552	0.2317	0.0128								
Concrete	0.0045			0.5126					0.0152	0.0355	0.3603	0.0579	0.0137

**Table 5:** Typical distances of the 3 detectors used in experiments and simulations in order to irradiate the cells placed as described in Fig. 2.

Tally region	Distances [cm]	
	From X-ray tube exit window	From X-ray tube focal spot
1(B)	0	15.5
2(B)^a^	14	29.5
3(B) ^a^	41	56.5

^a^Tally regions 2(B) and 3(B) are related to the Petri dish positions. Tally region 1(B) is referred to a numerical detector. See Fig. 2b for geometry details.

About the eye geometry implemented into the model, we referred to the classical works by Bherens^18,19^ and El Basha^20^ that can be considered the state of the art: the resulting geometry has been described in Fig. 2c. The material’s characteristics (Tables 1, 3), properly tuned for a Monte Carlo transport simulation, have been set accordingly to the above cited references.

However, the Monte Carlo simulation results have been investigated just to establish some general energy deposition guidelines without entering into the details of the radiation biological effects on the various eye components (e.g. the biological changes as a result of the radiation interaction with biomolecules).

### Scenario A—angiographic equipment

**Figure 3:**
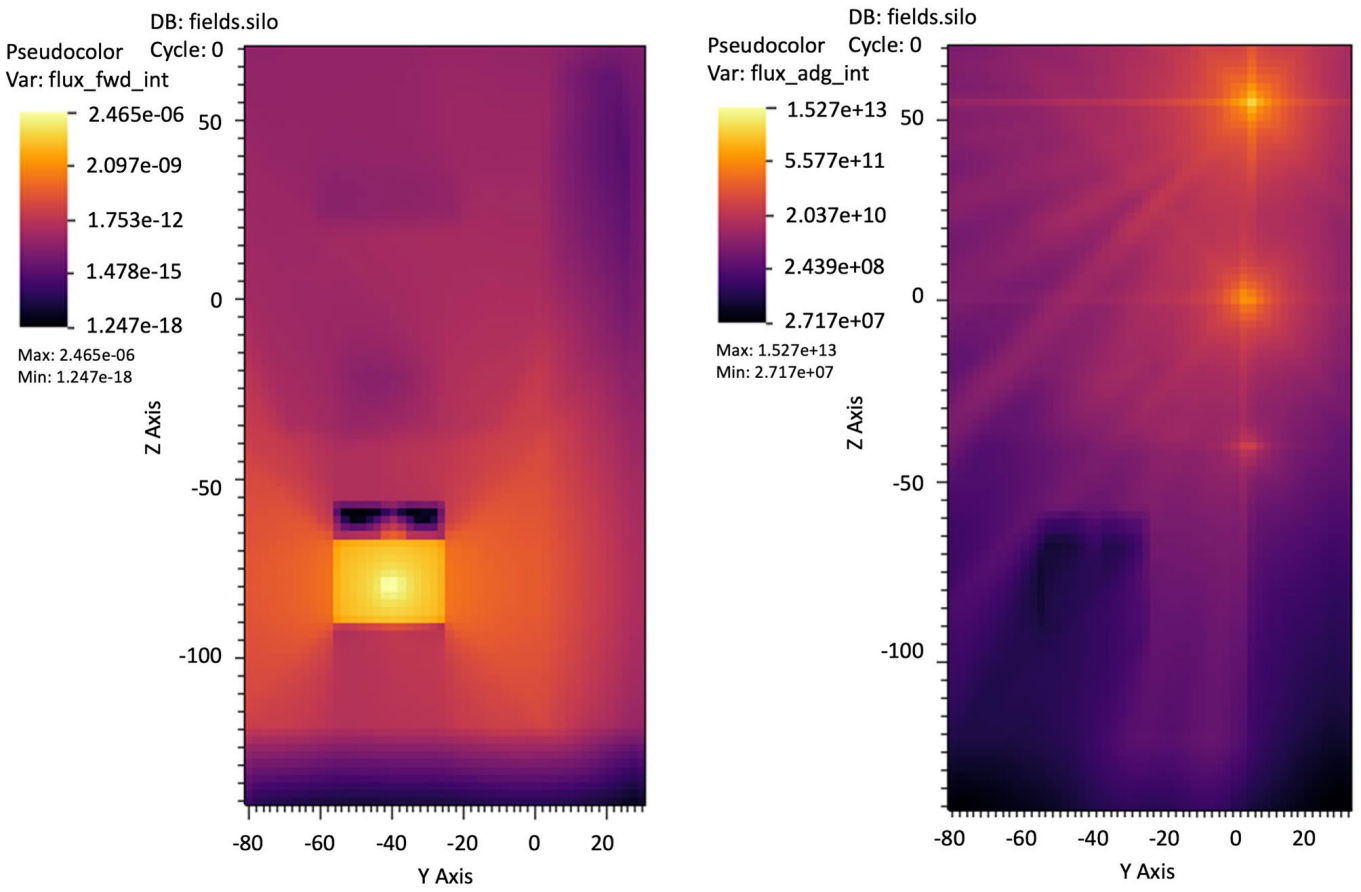
Typical Weight Window generation chain for MCNP, calculated with the ADVANTG discrete ordinates code. Left, forward flux values map interpreted by ADVANTG from the MCNP input file used as basis or the WW creation. Right, adjoint flux reflecting the importance function of the problem. ADVANTG simulations on a rectangular mesh of 5.0E+ 05 elements, Legendre polynomial order for scattering anisotropy modeling equal to 3, 8azimuthal and polar angles per octant, DPLUS MG photon library^24^. See Fig. 2a–c for geometry details.

**Figure 4:**
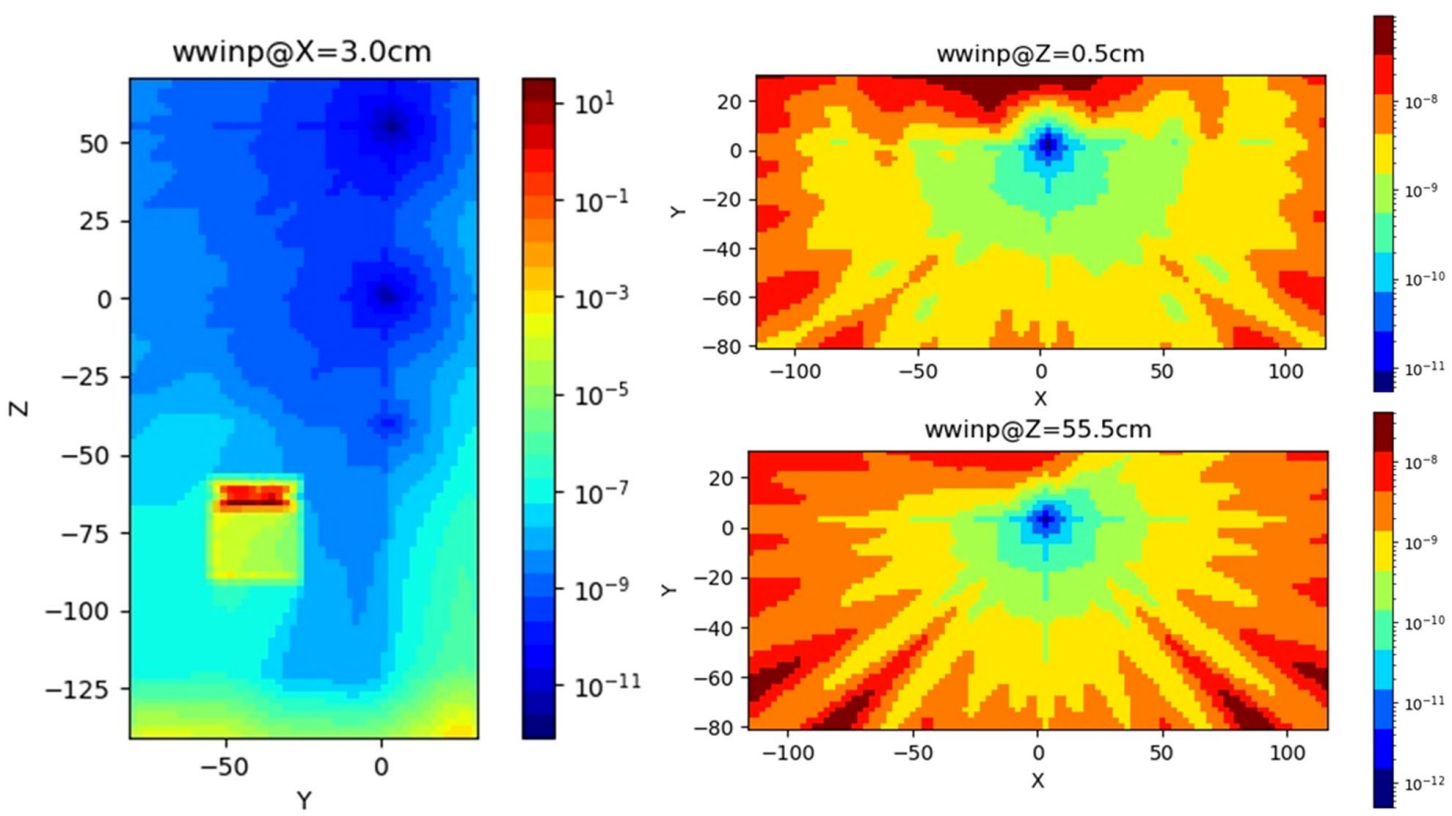
Typical WW distributions. Left, YZ view. Right-up, XY view, chest detector quote (2A). Right-low, XY view, eye lens quote (left eye, 1A). The slight asymmetry is due to the source centered in the vertical axis of the reference system and eye lens shifted by 3.5 cm. See Fig. 2a–c for geometry details.

The first case scenario (cells in the diffused photon field) has been built in MCNP6 for simulating the experimental conditions in which the cells have been irradiated and it is shown in Fig. 2a–c (see also Fig. 1). MCNP6 has been used for the spectrum estimation at the detector positions and at the eye lens (eye geometry fully implemented and introduced in a water phantom modeled following the International Standard ISO indication^21^), as reported in Fig. 2a–c, using a combination of the F4 tally (fluence, in cm^−2^) with the E option (cutoff energy set to 1 keV, maximum energy set to 100 keV, 23i tally option energy binning). The problem type has been set with the mode card with the P parameter. The photon cutoff energy has been chosen to be 1 keV with the physics options and a maximum energy of 100 keV has been set (phys card in the MCNP6 jargon). The simulations ran until a Relative Error (RE) less than 0.1 was reached for each estimated spectrum. In Tables 1, 2, 3 and 4 the material compositions^22^ and the parameters chosen for the Monte Carlo simulations are carefully described. The importance function (from the adjoint flux)^23–25^ used for the variance reduction biasing applied to the MCNP6 model is shown in Fig. 3 and has been generated by the tool ADVANTG along a CADIS (Consistent Adjoint Weight Importance Sampling) approach standing on the discrete ordinate transport code DENOVO^26^. The applied bias turns out unavoidable due to the dramatic dimensional difference between the whole source field and the goal of a reliable dose deposition in the eye and the eye lens. This approach partly overcome the warnings about Monte Carlo codes rose by Ainsbury in his review work^7^. A typical behavior of the weight window is shown in Fig. 4 obtained using the DPLUS MG photon library^27^. Due to the adjoint importance functions, described by the shown weight window derived by the ADVANTG code and applied to the model, the simulations run in a non-analog mode (biased simulations) in such a way to obtain acceptable results in an optimized simulation time. As order of magnitude, the Figure of Merit (FOM) has been found to be 10–100 times higher with respect to the correspondent analog simulations. It should be recalled that the FOM (proportional to RE^−2^ T^−1^) scaling up with the same RE means obtaining an equally scaled down simulation time or, alternatively maintaining the simulation time, dramatically reduce the RE. This proved an impressive increased simulation efficiency. The Weight Window has been set with the Weight Window Parameter (WWP) card applied to the photon transport and the source has been biased with the Source Bias (SB) card.

### Scenario B—research X-ray tube

**Figure 5:**
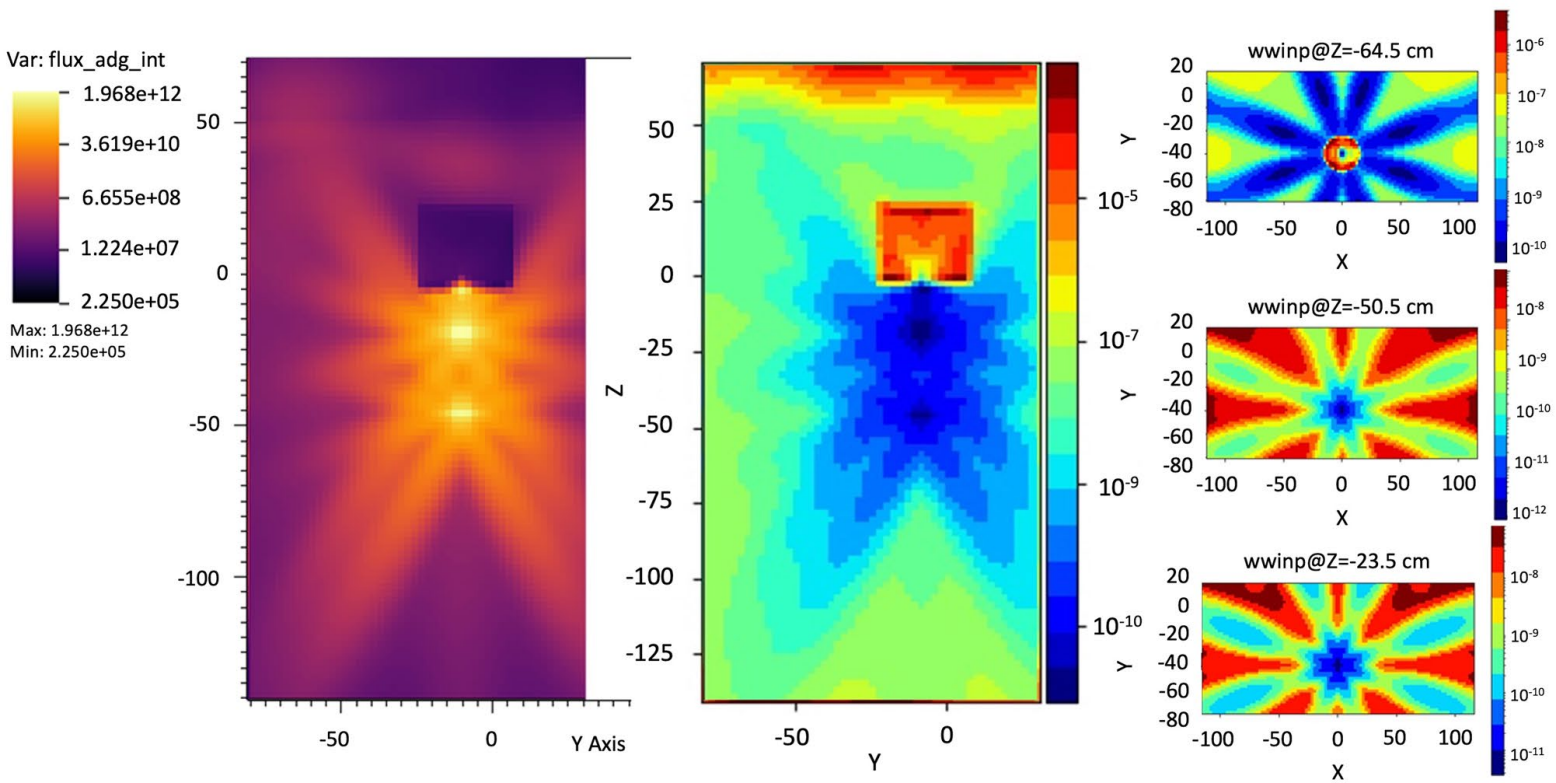
Importance function evaluated by the ADVANTG tool. Left, adjoint flux. Centre, Weight Window typical behavior in YZ view. Right, WW at the detector levels in XY view. ADVANTG simulations on a rectangular mesh of 5.0E+05 elements, Legendre polynomial order equal to 1 (linear anisotropy), 2azimuthal and polar angles per octant, DPLUS multigroup photon library^24^. See Fig. 2b for geometry details.

The second case scenario (cells inside the primary beam at different distances from the source) is presented in Fig. 2b (in position 1, a detector for the photon spectrum estimation directly outside the X-ray system has been implemented in MCNP; positions 2 and 3 representative of the Petri dishes). The spectra have been evaluated in the different positions for a 100 kV of each system using the F4 tally in combination with the E option card with a fine energy binning from 1 to 100 keV (bin width equal to 3.34 keV). The variance reduction has been demanded to the WW technique in combination with WWP and SB card. The WW has been generated by the ADVANTG tool as shown in Fig. 5. The normalized energy deposition has been evaluated for the three different detectors in order to estimate the relative differences in term of absorbed dose received by the different samples using an F6 tally evaluated on a single energy bin on all over the X-ray energies. Details on the detector position with respect to the X-ray device are shown in Table 5.

### Statistical analysis

Welch’s t-test was used to compare MN, BNC and p21-positive cells frequencies of the samples irradiated with different doses. The spectrum agreement between eye and Petri’ position was evaluated using the gamma-index (γ-index) analysis (0.03 keV/0.02). An agreement > 90% was considered adequate due to the spatial resolution of MC calculation.

The level of significance was established at P < 0.05.

### Cells and culture conditions

Human Lens Epithelial Cells B3 (HLE-B3, ATCC, USA) at low passages (5–7) were grown in Eagle’s minimal essential medium with Earle’s salt (Euroclone, Italy) supplemented with 20% fetal bovine serum (Euroclone, Italy), with 10,000 units/mL penicillin and streptomycin 10 mg/mL (Biological Industries, Israel), and 1% non-essential amino acid (Euroclone, Italy). Cell cultures were maintained in a humidified incubator at 37 °C, with 95% relative humidity and 5% CO_2_. For each timepoint of the following techniques, at least 3 independent replicates were used.

### Cytokinesis-blocked micronucleus assay

Twenty-four hours before irradiation, some 5 × 10^4^ cells were seeded on slides inside 35-mm Petri dishes. Immediately after irradiation, cytochalasin-B (final concentration 3 μg/mL in dimethyl sulfoxide, Sigma-Aldrich, St. Louis, MO, USA) was added. After 24 h, slides were fixed with cold methanol and stained with DAPI (4′,6-diamidino-2-phenylindole, Sigma-Aldrich) diluted in antifade solution (Vector Laboratories) to a final concentration of 1 μg/mL. Micronuclei (MN) were scored at 63× magnification using an Axiophot Z2 microscope with ultraviolet light (359 nm excitation filter, 441 nm barrier filter). Criteria for scoring MN were assumed following the approach of Fenech et al.^28^. MN frequencies were assessed scoring 1000 binucleated cells (BNC) for each replicate. The percentage of binucleated cells was evaluated scoring 1000 cells (mono- and binucleated).

### Immunofluorescence staining with p21 antibody

Twenty-four hours before irradiation, 5 × 10^4^ cells were seeded on slides inside 35-mm Petri dishes. Three and 24 h after irradiation, slides were fixed in cold methanol for 30 min and then incubated with p21 antibody (Santa Cruz) for one hour at 37 °C, followed by incubation with Alexa488 anti-mouse secondary antibody (Invitrogen) and counter-stained with DAPI. One thousand cells per replicate were scored on random fields at 63× magnification with an Axiophot Z2 microscope, assessing the frequencies of p21-positive cells.

## Results

### MC and dosimetric results

**Figure 6:**
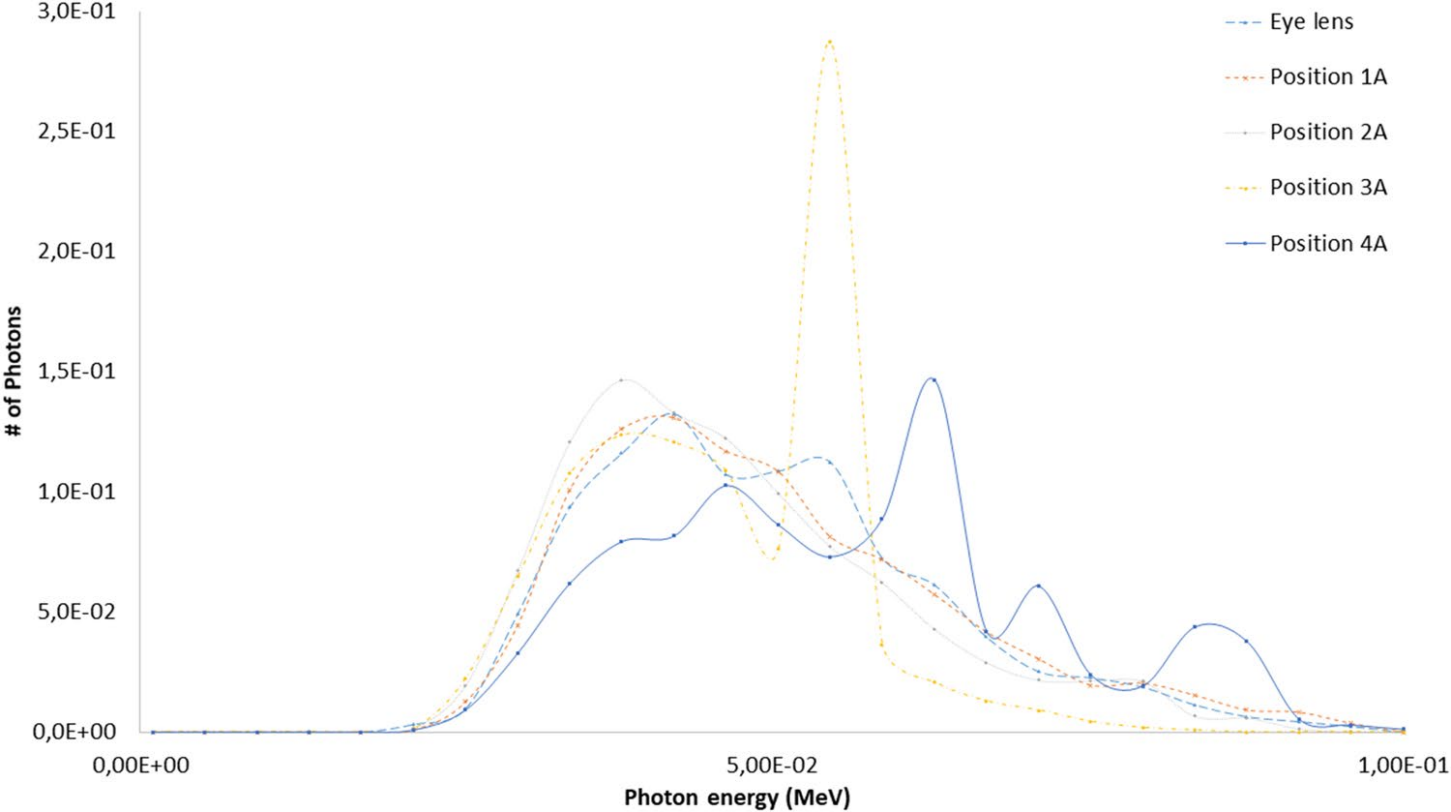
Scenario A. Photon spectra at the detectors (see Fig. 2 for details of position and characteristics) estimated with the MCNP code with the 100 kV endpoint X-ray source (see Fig. 2 for details). Photon energy scale in MeV.

**Figure 7:**
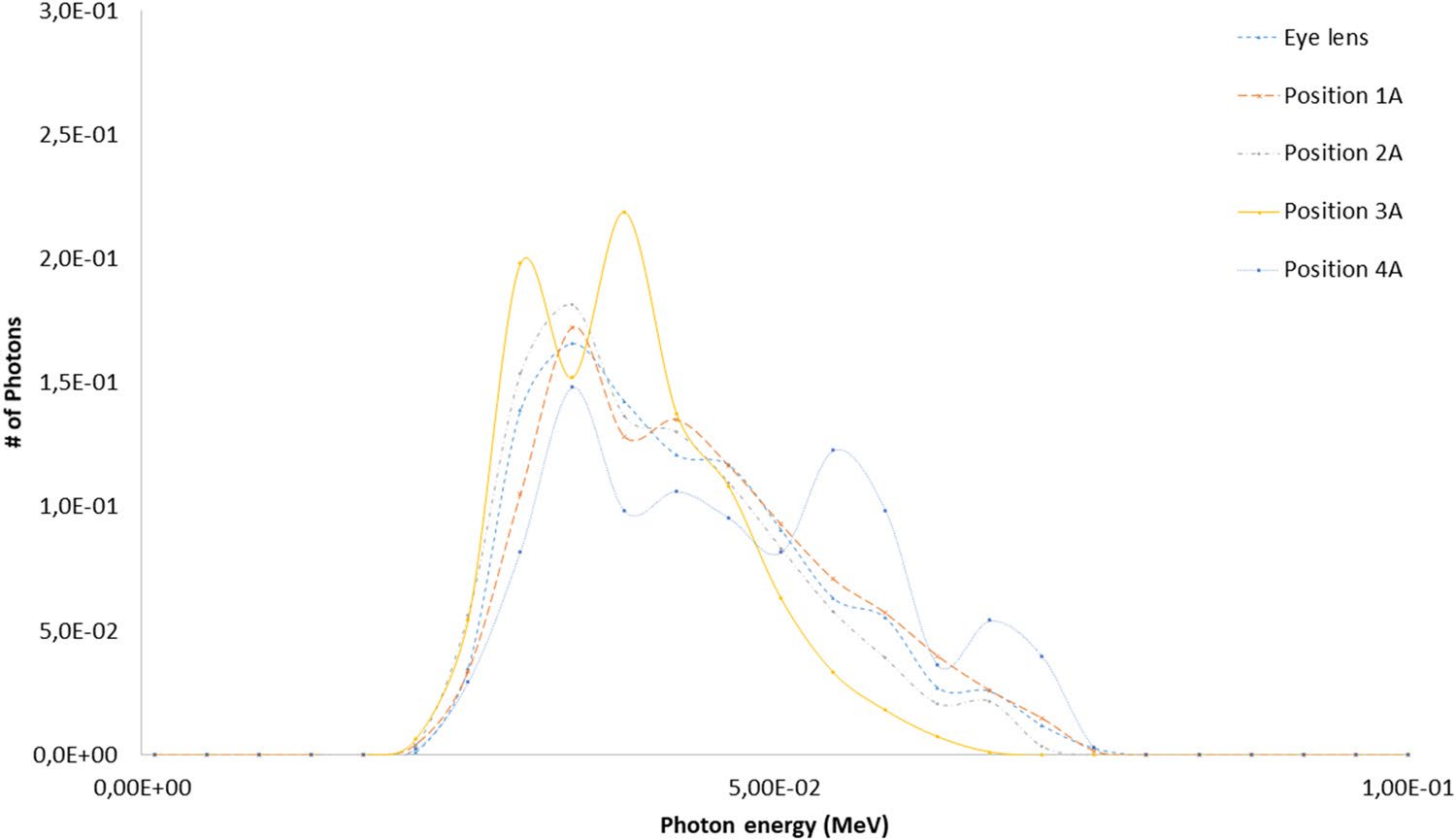
Scenario A. Photon spectra at the detectors (see Fig. 2 for details of position and characteristics) estimated with the MCNP code with the 80 kV endpoint X-ray source. Photon energy scale in MeV.

**Figure 8:**
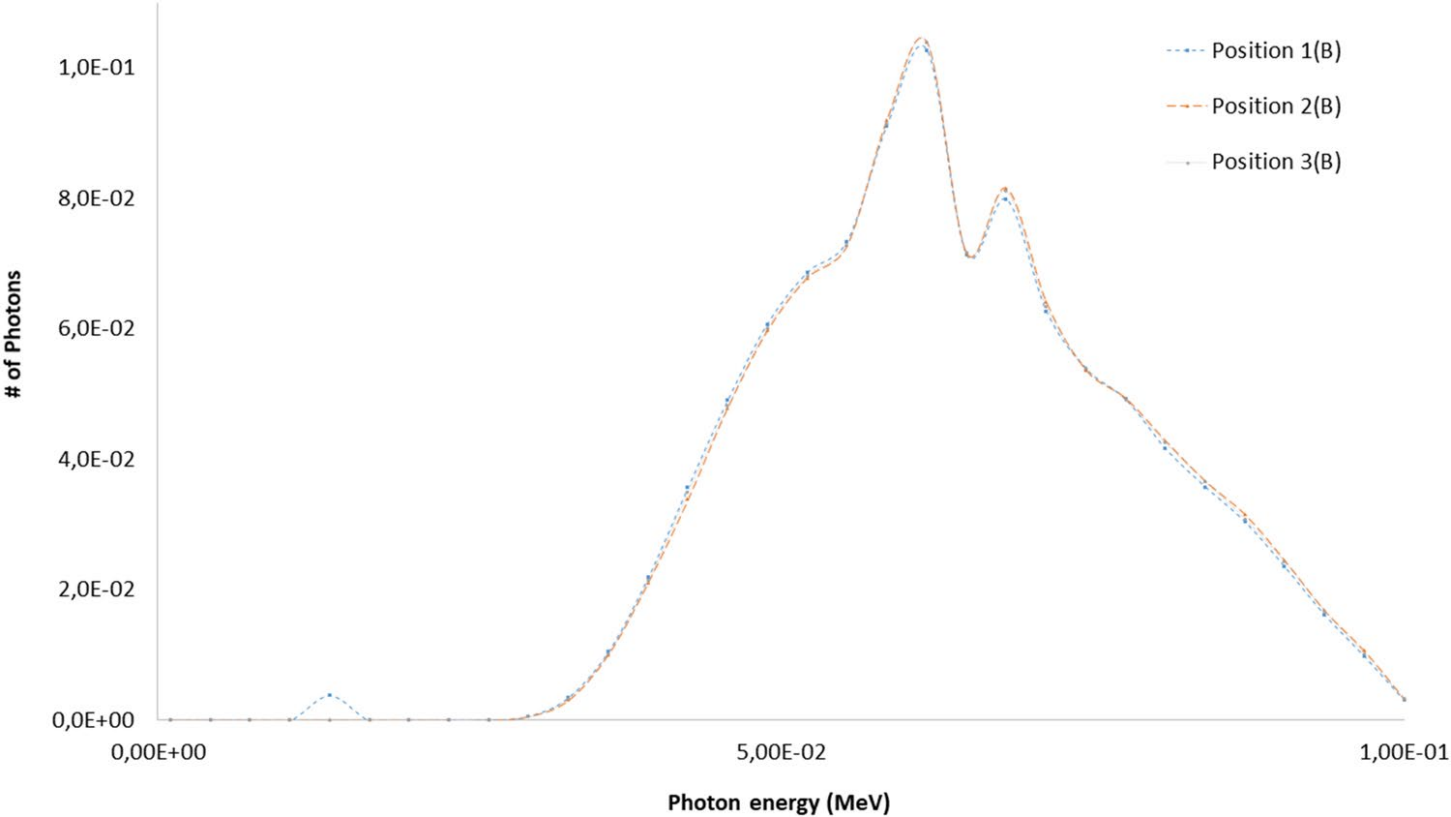
Scenario B. Photon spectra evaluation at the three MCNP detectors (see Fig. 2 and Table 5 for details). Photon energy scale in MeV.

**Figure 9:**
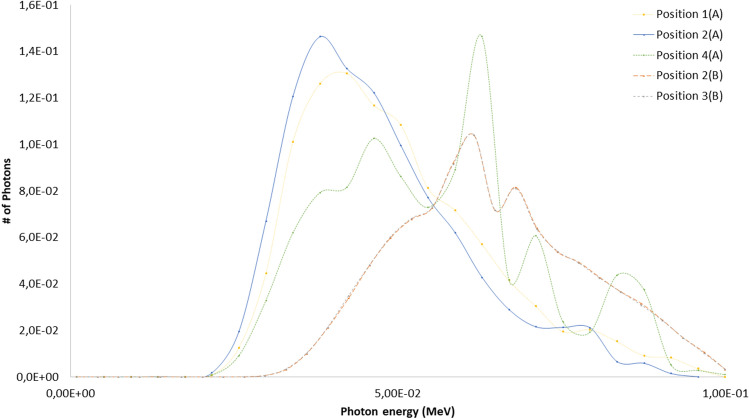
Scenario A and B spectral comparison at the various Petri dishes as estimated with MCNP (see Fig. 2, and Tables 1, 2, 3, 4, 5 for details). Photon energy scale in MeV.

The eye lens, Petri dishes and numerical detectors collocations for the A and B scenarios already described are resumed in Table 6 (see Fig. 2 for details). The results related to the A and B scenario are reported in Figs. 6, 7, 8, 9 and Tables 7 and 8. The spectra estimated with the MCNP6 code for A scenario and the two sources are shown in Fig. 6 and 7. The Petri dishes have been simulated as in the experimental conditions in the positions 1, 2 and 4 (A scenario). The spectra estimated with MCNP6 for the B scenario are shown in Fig. 8. The Petri dishes have been simulated as in the experimental conditions in positions 2 and 3 (B scenario). The relative absorbed dose for the 3 detectors of the B scenario is shown in Table 7, normalized for particle source and exposure time. The comparison between the spectra, as estimated with MCNP6, in both scenarios in the Petri dish positions, is shown in Fig. 9 and Table 8.

**Table 6:** Eye lens (*), Petri dish (X) and numerical detector (O) collocation in the different scenarios (see Fig. 2 for details).

Detector position	Detector type in scenarios	
	A	B
Eye lens	*	
1	X	O
2	X	X
3	O	X
4	X

**Table 7:** Relative absorbed dose fraction values per unit of exposure time at the detectors of the B scenario (see Fig. 2 and Table 5 for details).

Tally region	Relative absorbed dose	RE
1(B)	1.00E+00	0.0008
2(B)	2.53E−01	0.0012
3(B)	6.81E−02	0.0032

RE, relative error.

**Table 8:** Scenario A and B spectral main characteristics at the Petri dishes positions in scenarios A and B.

	1(A)	2(A)	4(A)	2(B)	3(B)
Mean	4.98E−02	4.71E−02	5.57E−02	6.42E−02	6.43E−02
Median	1.96E−02	2.12E−02	3.29E−02	2.28E−02	2.27E−02

The investigated doses on the LEC placed on the couch over the patient-like phantom, on the phantom thorax and on the eye positions were measured using Gafchromic films and ranged from 135 to 300 mGy using a clinical equipment, while those investigated with the research system ranged from 25 to 300 mGy (different doses were obtained with different exposure durations, while the two dose-rates were obtained moving the position of the cell support from 29.5 to 56.5 cm from the X-ray source). The dose-rates investigated using the clinical radiological equipment were lower than those used for research purposes thanks to the specific features of the angiographic system that is optimized for reducing the dosage to the workers in the clinical practice.

**Figure 10:**
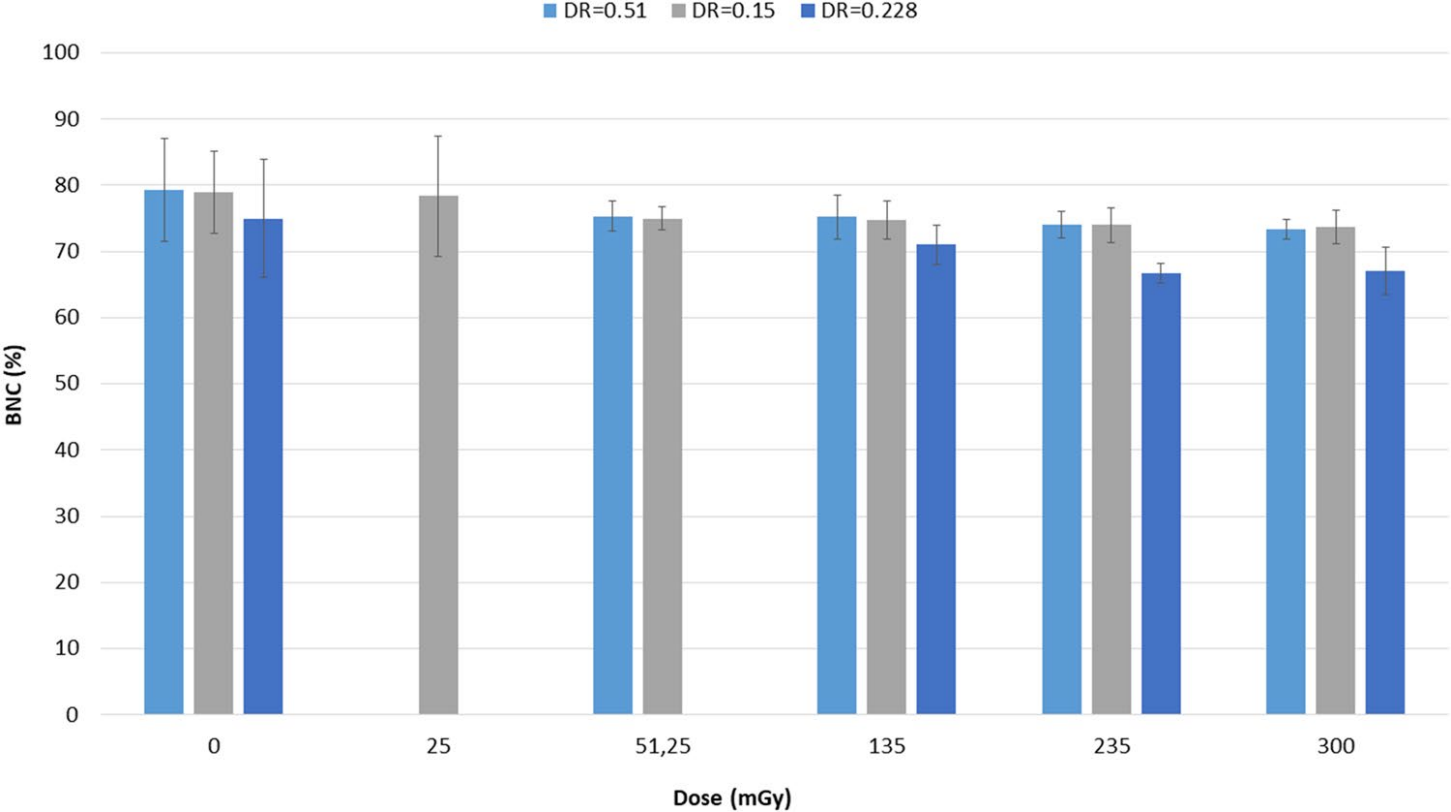
Frequencies of binucleated cells (BNC). In all irradiated samples the frequencies of BNC were lower than the unirradiated controls although not statistically significant. Dose rate (DR) is expressed in Gy/min.

All irradiated samples showed frequencies of BNC lower than the unirradiated controls (Fig. 10). However, the differences were not statistically significant. Similarly, no effects on cell viability were observed at the studied doses.

**Figure 11:**
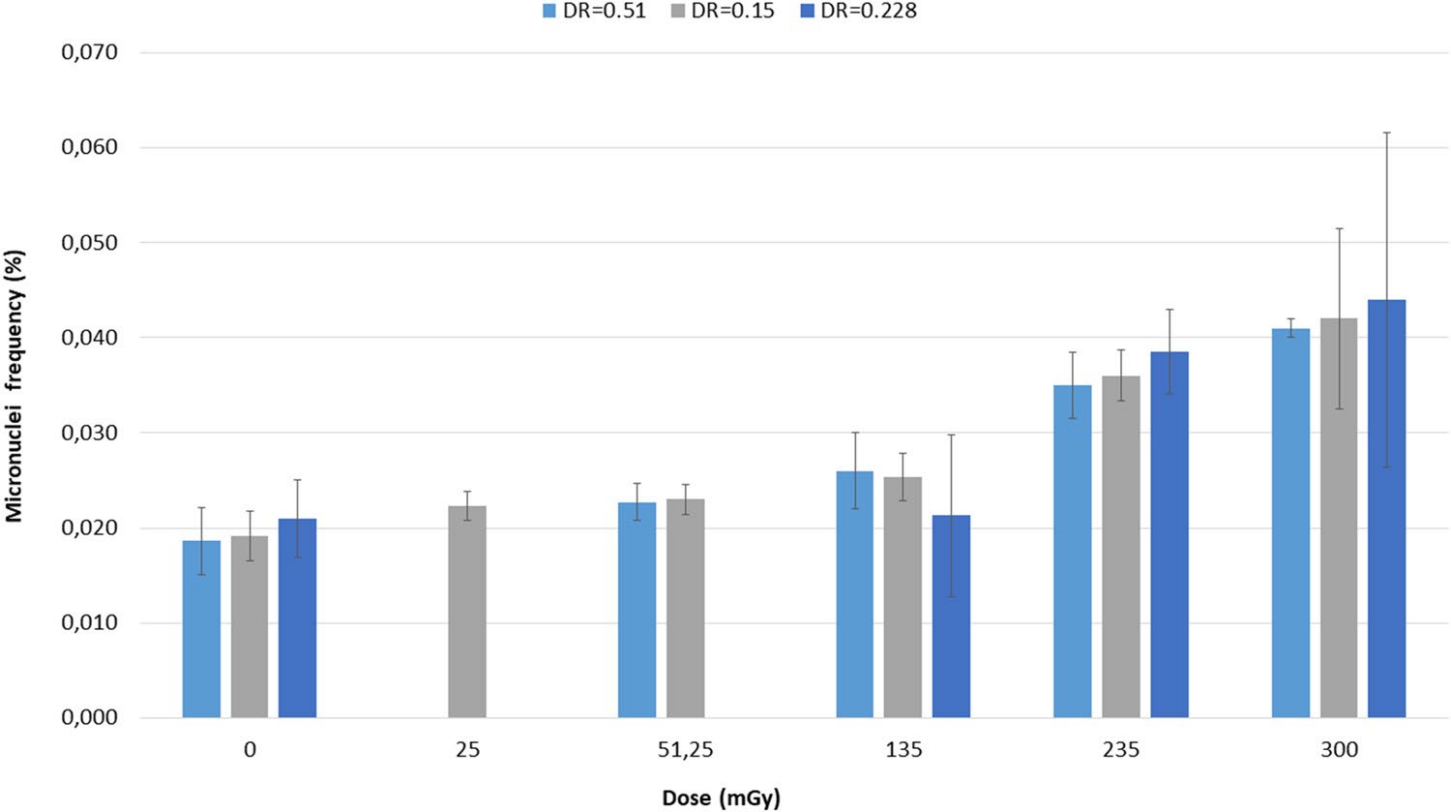
Frequencies of micronuclei (MN) in BNC. Dose rate (DR) is expressed in Gy/min.

All irradiated samples showed frequencies of MN higher than the unirradiated controls (Fig. 11). Cells irradiated with 25 mGy at 0.15 Gy/min showed a MN frequency significantly higher (p = 0.0276) than the respective control. Cells irradiated with 51.25 mGy at 0.51 and 0.15 Gy/min showed MN frequencies significantly higher (p = 0.0407 and p = 0.0069, respectively) than the controls. Cells irradiated with 135 mGy at 0.51 and 0.15 Gy/min showed MN frequencies significantly higher (p = 0.0242, and p = 0.0277, respectively) than the controls. Cells irradiated at 235 mGy with 0.51 and 0.15 Gy/min (scenario B) and with 0.228 Gy/min (scenario A) showed MN frequencies significantly higher (p = 0.0016, p = 0.0119 and p = 0.0075, respectively) than the controls. Cells irradiated at 300 mGy with 0.51 and 0.15 Gy/min (scenario B) and with the angiographic device (scenario A) showed MN frequencies significantly higher (p < 0.0001, p = 0.0472 and p = 0.0053, respectively) than the controls.

**Figure 12:**
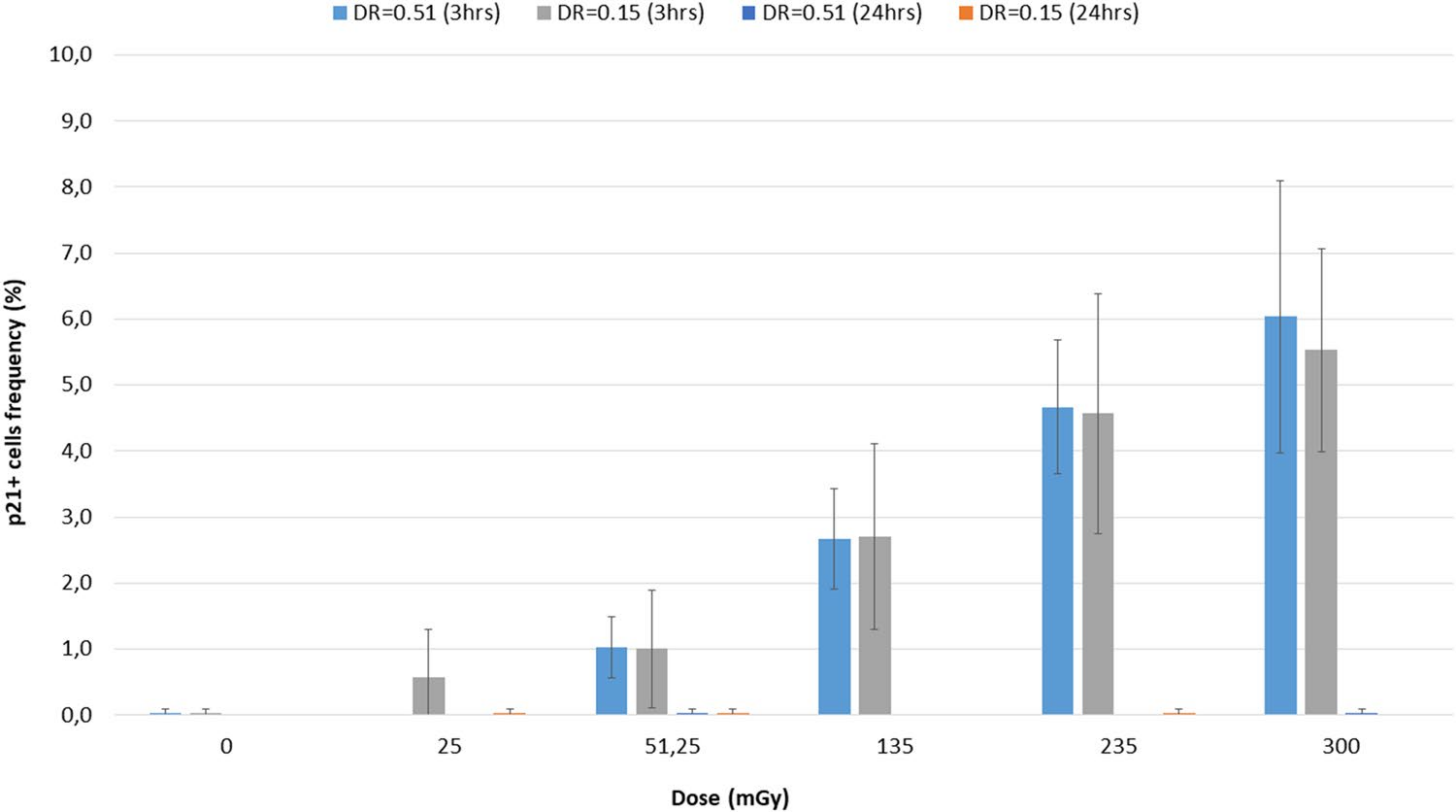
Frequencies of p21-positive cells. After 3 h, all irradiated samples showed frequencies of p21-positive cells higher than the unirradiated control. After 24 h, the level of p21-positive cells was the same as in unirradiated samples. Dose rate (DR) is expressed in Gy/min.

All irradiated samples showed frequencies of p21-positive cells higher than the unirradiated controls (Fig. 12). Cells irradiated with 51.25 mGy at 0.51 Gy/min showed frequencies of p21-positive cells significantly higher (p = 0.0233) than the controls. Cells irradiated with 135 mGy at 0.51 Gy/min showed frequencies of p21-positive cells significantly higher (p = 0.0266) than the controls. Cells irradiated with 235 mGy at 0.51 and 0.15 Gy/min showed frequencies of p21-positive cells significantly higher (p = 0.0156 and p = 0.0495, respectively) than the controls. Cells irradiated with 300 mGy at 0.51 and 0.15 Gy/min showed frequencies of p21-positive cells significantly higher (p = 0.0371 and p = 0.0251, respectively) than the controls. Twenty-four hours after irradiation, the level of p21-positive cells was the same as in unirradiated samples.

**Figure 13:**
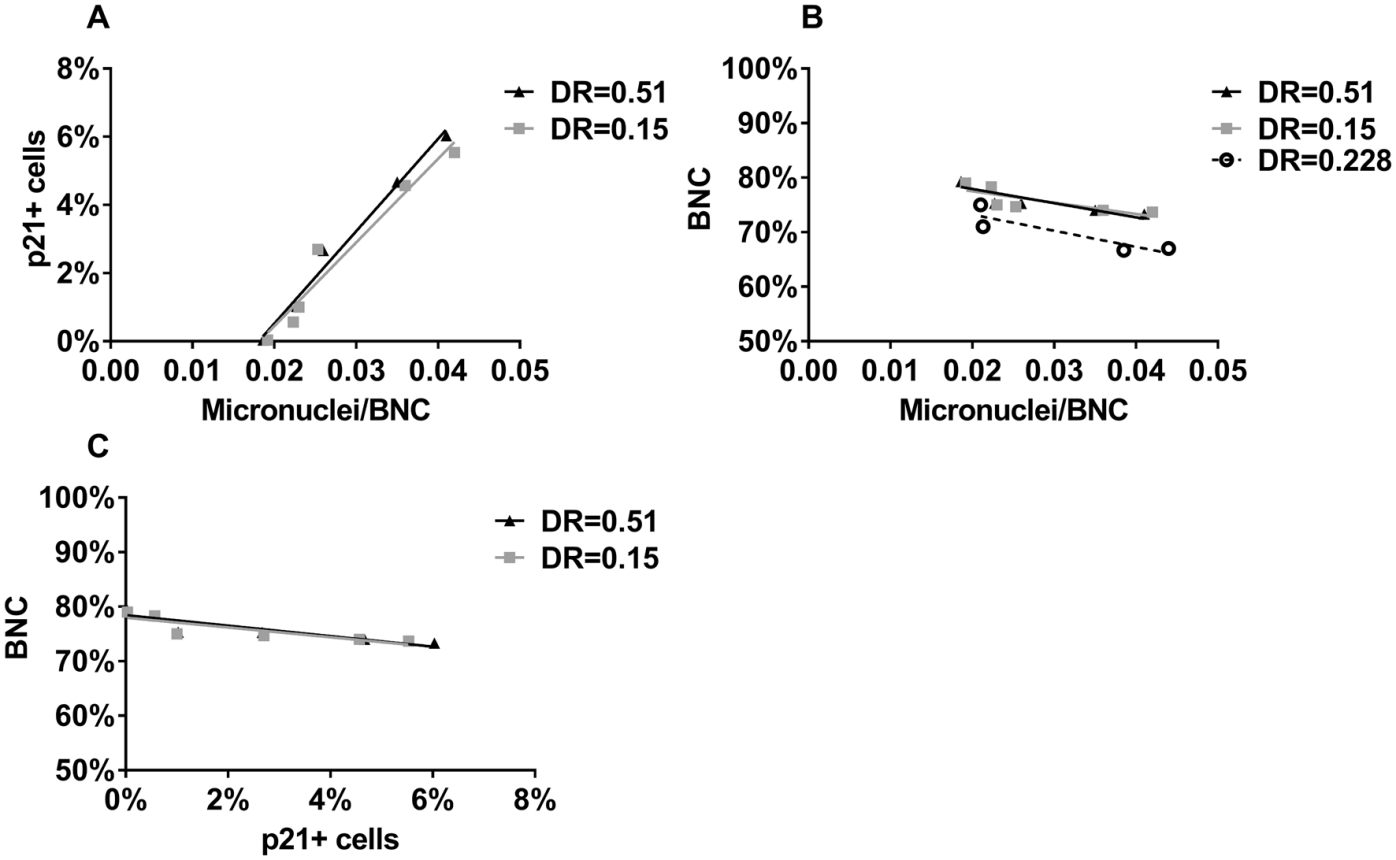
Frequencies of (**a**) p21-positive cells and BNC (**b**) versus frequencies of MN. Frequencies of (**c**) BNC versus those of p21-positive cells. Dose rate (DR) is expressed in Gy/min.

Correlations between frequencies of MN and p21-positive cells (Fig. 13A) were significant both for cells irradiated with 0.51 and 0.15 Gy/min (p < 0.0001, R^2^ = 0.85 and R^2^ = 0.77, respectively), and for pooled data (p < 0.0001, R^2^ = 0.80). Correlations between frequencies of MN and BNC (Fig. 13B) were not significant for cells irradiated with 0.51 and 0.15 Gy/min or with the angiographic equipment, but were significant for pooled data, although the correlation was poor (p = 0.0004, R^2^ = 0.22). Correlations between frequencies of p21-positive cells and BNC (Fig. 13C) were not significant for cells irradiated with 0.51 and 0.15 Gy/min but were significant for pooled data although the correlation was poor (p = 0.0106, R^2^ = 0.16).

## Discussion

Eye lens dose from occupational exposure depends on the specific exposure conditions already widely investigated in literature. However, the impact of incident energy spectrum, dose and dose-rate on LEC are still to be clarified using research and clinical radiological equipment.

The incident energy spectrum on the operator eye lens emerging from the patient/phantom is obviously substantially degraded respect to the incident one and low energy component might negatively increase on the ionizing radiation induced effects. In this context, Monte Carlo simulation has allowed us to investigate the modification of the radiation spectrum and to compare a clinical machine (occupied for diagnosis and treatments) and a research machine dedicated to experiments, supporting the interpretation of results. More in details the incident spectrum on the Petri are similar enough. The low energy component which could have increased the RBE value is negligible given the size of the cells, the presence of the support and the culture liquid. The absorbed spectrum in the Petri dishes was in good agreement with the one calculated for the eye lens (γ-index > 95%) for scenario A. Similarly, there were a good agreement (γ-index > 90%) in the absorbed spectrum at the position 2 and 3 of scenario B.

Moreover, the speed up obtained thanks to the physical bias of the photon transport process thanks to the CADIS tool, let us envisage a quasi-routinely analysis approach.

In addition, the dose rate varies in the investigated positions thus appropriate conversion factor to be based on radiobiological models using LEC are mandatory. In addition, the use of Gafchromic films permits to check the delivered dose at each irradiation and being irrespective of incident spectrum to calculate the dose in several points and in different experimental conditions (e.g. scenario A and B).

In this context, our experimental setup allows the characterization of radiation induced effects on LEC taking into consideration several biological pathways using both a research and a clinical equipment.

The application of the MN test with LEC B3 proved to be a very sensitive model to detect DNA damage. It is usually said that the lowest dose of ionizing radiation that is detectable with the MN test is 200–300 mGy^29^. However, this threshold is based on studies employing lymphocytes^30^, whereas Boei et al.^31^ found a significantly increase of micronuclei in human fibroblasts even at doses as low as 20 mGy. Cell-specific differences in the sensitivity for the MN test may be due to differences in the efficiency of the DNA repair systems and/or in the stringency of the DNA damage checkpoints^32^.

In our study, we found that DNA damage was induced even at low, occupationally relevant doses (Fig. 11). This DNA damage induced a transient increase of p21, as detected after 3 h, but completely disappeared after 24 h (Fig. 12). The induced DNA damage and transient increase of p21 was not sufficient to exert a significant effect on proliferation (Fig. 13). Similarly, cellular senescence (assayed by β-Galactosidase activity) was not detected (data not shown).

These results may provide a hypothesis on the mode of action of low doses of ionizing radiation in the onset of cataract.

It is widely accepted that genomic damage of LEC is a key mechanism of cataractogenesis^33^. Indeed, it has been showed that mice homo- and heterozygous for the DNA repair genes *ATM*, *BRCA1* and *RAD9* develop cataracts earlier and in greater numbers^7,8,34^. A further confirmation of this hypothesis can be seen in the fact that patients with the well-known DNA repair disorders, such as Trichothiodystrophy, Cockayne, Rothmund-Thomson and Werner syndromes^9^ are prone to develop cataracts. It is noteworthy that we found genomic damage even at doses as low as 25 mGy. This is in agreement with the results of Markiewicz et al.^35^, who found DNA damage in LEC of mice exposed to 20 mGy and should be considered by radio-protectionists.

Unrepaired DNA damage usually triggers two cellular responses: apoptosis or cell cycle block. The relationship between apoptosis and cataractogenesis is controversial: some studies provided evidence that apoptosis is not observed in cataractous lenses^36^, while other investigators have found apoptotic LEC in cataractous lenses and have suggested that this to be a cause of cataract formation^37^. These discrepancies may be due to different aetiologies (induced by radiation, age, diabetes, etc.) of the different types of cataract (nuclear sclerotic, cortical, posterior subcapsular). Using low doses of X-ray, we found no effects on cell vitality and proliferation. To this, we would add that Ainsbury et al.^33^ found an increased resistance to apoptosis in *Atm*^*−*^*/Brca1*^*−*^ mice, despite the fact that these were cataract-prone (as said before). We would therefore opt for the hypothesis that apoptosis is not responsible for cataract induced by low doses of X-ray.

**Figure 14:**
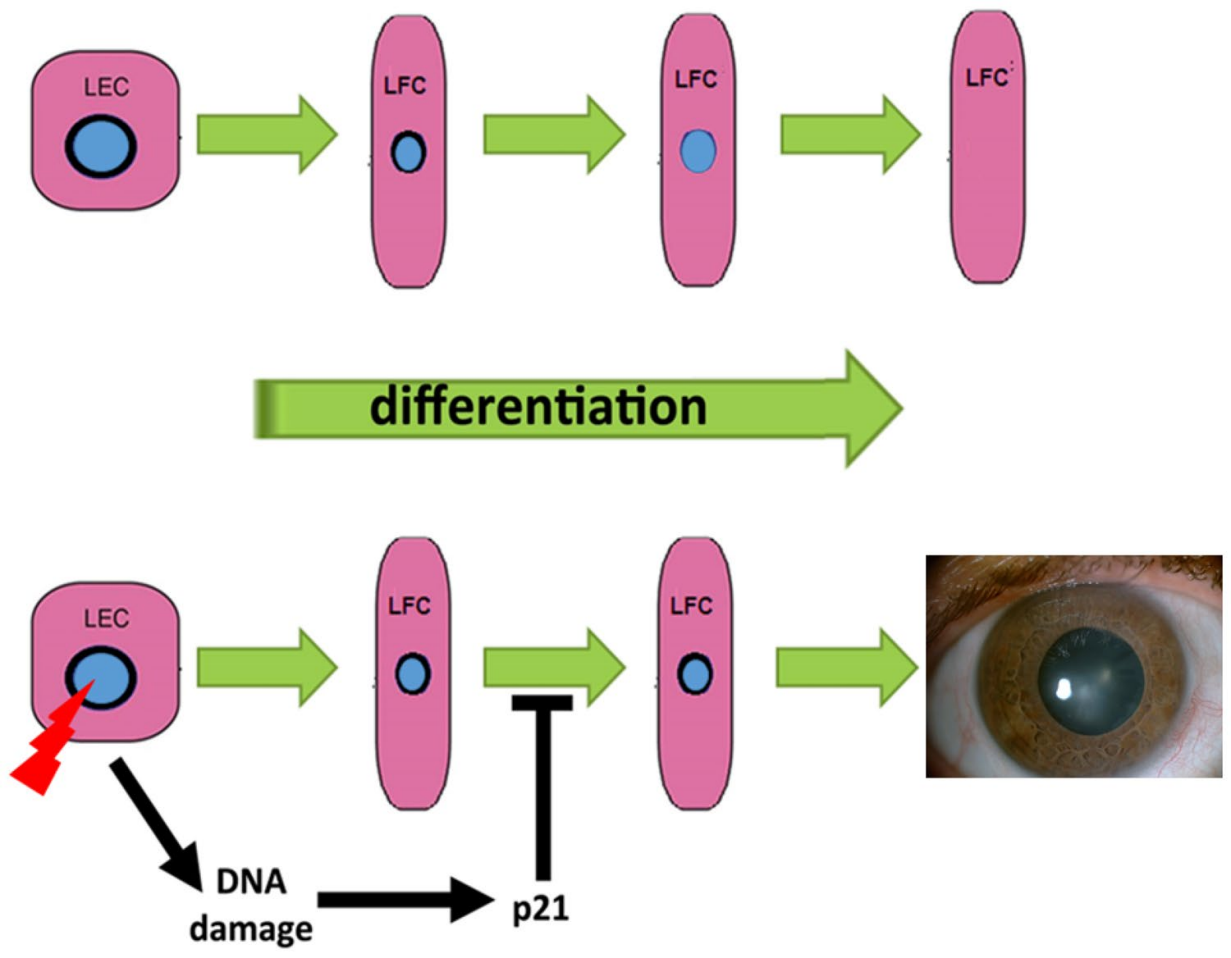
Proposed model of DNA damage-induced cataract. In the upper panel, the normal process of differentiation from LEC to lens fiber cells (LFC) comprises loss of the nuclear envelope (in black) and consequent degradation of the nucleus (in light blue color). In the lower panel, X-ray-induced DNA damage causes an increase of p21, which impedes the disassembly of the nuclear envelope, thus impairing differentiation and leading to cataract formation.

The second response to unrepaired DNA damage is cell cycle block. DNA double strand breaks trigger the expression of the cyclin-dependent kinase inhibitor p21 (encoded by the *CDKN1A* gene), in a p53-dependent and/or independent manner^38^. The prolonged expression of p21 determines a permanent cell cycle arrest known as senescence^38^. This is the case, for example, of cells irradiated with high doses, while at lower doses the induction is transient^39,40^. In agreement with this, we found a transient increase of p21 in irradiated LEC that could be not enough to induce neither senescence nor cellular death. This finding may have a relevant importance, since this induction of p21 may interfere with the disassembly of the nuclear envelop in differentiating LEC, leading to cataract formation^41^ (Fig. 14). This in in agreement with findings that linked the p21 increase with cataractogenesis^42,43^.

Indeed, lack of enucleation is one of the features of aberrant lens fiber cells in cataractogenesis^44,45^. Further studies in this direction could better clarify the relationship we suggested between DNA damage, transient p21 induction and the inability of LEC enucleation.

## Conclusion

Our results support the hypothesis that the induction of transient p21 may interfere even at low doses of X-rays with the disassembly of the nuclear envelop in differentiating LEC, leading to cataract formation, while the apoptosis seems not responsible for this effect.

Further studies are mandatory to better clarify our findings, i.e. the relationship we suggested between DNA damage, transient p21 induction and the inability of LEC enucleation.

Another aspect that needs further investigation is the role played by differences in the radiation quality. While we saw little biological differences due to variations of the dose rate (0.51 vs 0.15 Gy/min under scenario B), the set up exposure condition (scenario A vs. B) seems more important. Thus, clarifying the biological effects of different spectra could be useful also in the radioprotection field.
